# Five Indigenous Plants of Pakistan with Antinociceptive, Anti-Inflammatory, Antidepressant, and Anticoagulant Properties in Sprague Dawley Rats

**DOI:** 10.1155/2017/7849501

**Published:** 2017-11-23

**Authors:** Hammad Ismail, Ammara Rasheed, Ihsan-ul Haq, Laila Jafri, Nazif Ullah, Erum Dilshad, Moniba Sajid, Bushra Mirza

**Affiliations:** ^1^Department of Biochemistry and Biotechnology, University of Gujrat, Gujrat 50700, Pakistan; ^2^Department of Biochemistry, Quaid-i-Azam University, Islamabad 45320, Pakistan; ^3^Department of Pharmacy, Quaid-i-Azam University, Islamabad 45320, Pakistan; ^4^Department of Biochemistry, Bahauddin Zakariya University, Multan 60000, Pakistan; ^5^Department of Biotechnology, Abdul Wali Khan University, Mardan, Pakistan; ^6^Department of Bioinformatics and Biosciences, Capital University of Science and Technology, Islamabad, Pakistan

## Abstract

Five medicinal plants of Pakistan were investigated for their antinociceptive, anti-inflammatory, antidepressant, and anticoagulant potential. Antinociceptive activity was estimated by hot plate and writhing assay. In hot plate assay,* Quercus dilatata* (52.2%) and* Hedera nepalensis* (59.1%) showed moderate while* Withania coagulans* (65.3%) displayed a significant reduction in pain. On the other hand, in writhing assay,* Quercus dilatata* (49.6%),* Hedera nepalensis *(52.7%), and* Withania coagulans* (62.0%) showed comparative less activity. In anti-inflammatory assays crude extracts showed significant edema inhibition in a dose dependent manner. In carrageenan assay, the highest activity was observed for* Withania coagulans *(70.0%) followed by* Quercus dilatata* (66.7%) and* Hedera nepalensis* (63.3%). Similar behavior was observed in histamine assay with percentage inhibitions of 74.3%, 60.4%, and 63.5%, respectively. Antidepressant activity was estimated by forced swim test and the most potent activity was revealed by* Withania coagulans* with immobility time 2.2s (95.9%) followed by* Hedera nepalensis *with immobility time 25.3s (53.4%). Moreover, the crude extracts of* Fagonia cretica *(74.6%),* Hedera nepalensis* (73.8%), and* Phytolacca latbenia* (67.3%) showed good anticoagulant activity with coagulation times 86.9s, 84.3s, and 67.5s, respectively. Collectively, the results demonstrate that these five plants have rich medicinal constituents which can be further explored.

## 1. Introduction

Since the beginning of human civilization medicinal plants have been used for therapeutic purposes against different ailments; therefore they are considered to be one of the oldest form of human healthcare known to date [1]. It is also a matter of fact that medicinal plants and their products contribute more than half of all clinically administered drugs of modern day. According to an estimate ~40% of new drugs approved during last two decades were of natural origin [2]. More precisely, between 1983 and 1994, 39% of the 520 newly approved drugs were either natural products themselves or their derivatives and 40–80% of drugs developed against bacterial infections and cancer were of natural origin, which indicates that the use of natural products in treating human ailments has been making rapid progress and getting popular with the passage of time [3–7]. Therefore, plants and their products not only hold a substantial position in drug discovery but also can play a dynamic role in the revenue generation and improving the economic conditions of developing countries like Pakistan [1].

Fortunately, Pakistan is gifted with a rich flora of almost 5700 species of which around 2000 are reportedly medicinal plants. It is also matter of fact that, in Pakistan, over 40% of the population especially those living in rural areas are getting health care by local healers (Hakims), who prescribe herbal preparations [8]. However, the revenue generated by Pakistan is far lower than the trade in medicinal plants made by India and China recently [1]. This may be due to the lack of exploration of new medicinal plants or evaluation of the existing medicinal plants for their potential properties against different ailments. Therefore, based upon the knowledge from local healers and the available literature, five medicinal plants (*Quercus dilatata, Fagonia cretica, Hedera nepalensis, Phytolacca latbenia, *and* Withania coagulans*) were selected from different areas of Pakistan for the evaluation of antinociceptive, anti-inflammatory, antidepressant, and anticoagulant properties in Sprague Dawley rats.

*Quercus dilatata* Linn. is commonly called holly oak, green oak, or moru oak [9]. The tree produced galls which can be used against dysentery, chronic diarrhea, hemorrhages, and asthma [10]. This plant is also reported for its wound healing ability in rats and antioxidant enzymes activation [11, 12].* Fagonia cretica* Linn, family Zygophyllaceae [13] commonly known as “Dhamasa” in Pakistan, is used against thirst, vomiting, dysentery, typhoid, toothaches, and skin diseases [14]. There are other ailments for which this plant is reported to be effective, including but not limited to neck swellings, cancer [15], snake bite [16], diuretic, analgesic, antipyretic, antidote, antiseptic, tonic, bitter, antiasthmatic, stomachic, and stimulant [17]. It also contains antioxidants and can be used as blood purifier and against scabies [18–20].* Hedera nepalensis* K Koch. Is from Araliaceae family and popular as Himalayan Ivy or Nepalese Ivy [21]. In Pakistan, this plant is traditionally used against cancer and diabetes and as purgative [22, 23]. Some parts of this plant like leaves and berries are known to be stimulating, febrifuge, diaphoretic, antispasmodic, cathartic, and hypoglycemic [24, 25].* H. nepalensis* is also effective against fever, rheumatism, and pulmonary infection [26]. The indigenous plant* Phytolacca latbenia* is an abundant resource in Pakistan, distributed across more than 13,200 sq km, from an elevation of 1500–3000 m in the Murree, Galyat, Swat, Dir, Kaghan, and Kashmir hills [27]. This species has been known for its anti-inflammatory activities and has been widely used as traditional medicine by local community in Pakistan. Phytochemical analysis showed that* P. latbenia* is rich in saponins and terpenoids [28, 29]. Oil from the root is also an important product which is used for the treatment of pain in joints. Root of this plant is also used for the production of a resinoid substance called phytolaccin, which is used in medicines and to dilute belladonna (http://www.tropicos.org).* Withania coagulans *(Stocks) Dunal belongs to family Solanaceae, commonly known as paneer (cheese maker) in Punjab [30]. This plant has been very popular amongst local healers in Pakistan. Its fruits are applied in folk medicine on wound and are used in asthma. Fruits are also reported to be diuretic, hypoglycemic, and hypolipidemic [31]. Other ailment in which this plant can be effective includes liver troubles, diabetes, dental problems, inflammations, and depression [31–37].

## 2. Methods

### 2.1. Plant Material

In the present study, five plants* Quercus dilatata*,* Hedera nepalensis*,* Fagonia cretica*,* Phytolacca latbenia,* and* Withania coagulans* were selected on the basis of their use in folk medicine. These plants were identified at the Department of Plant Sciences, Quaid-i-Azam University, Islamabad, Pakistan, by Professor Dr. Rizwana Aleem Qureshi (taxonomist). Their voucher specimen numbers were deposited in the “Herbarium of Medicinal Plants of Pakistan” in QAU Islamabad, Pakistan.

### 2.2. Extraction

Preparation of extracts was carried out by maceration. Briefly for* Q. dilatata*, leaves were taken while for the rest of the plants aerial part of the plants was taken and was dried under the shade. The plant material was then coarsely powdered and 200 g of it was soaked in 500 ml of solvent mixture containing methanol and chloroform (1 : 1). After five days, extract was filtered by using Whatman filter paper number 1. The filtrate was then concentrated with rotary evaporator at 40°C under low pressure. These dried crude extracts were named, respectively, with reference to plant and were stored at 4°C for further studies.

### 2.3. Preparation of Controls and Samples

Stock solution of each extract was prepared as 100 mg/ml while the standard drugs (Morphine, Aspirin, Fluoxetine HCl, Chlorpheniramine maleate, and diclofenac potassium) were prepared as 50 mg/ml in normal saline (0.9%). The extracts and standard drugs were administered orally as per rat's body weight. Three different concentrations of the extracts (200 mg/Kg, 100 mg/Kg, and 50 mg/Kg) were used to determine the potential effect of the plants while the standards drugs were administered as 10 mg/Kg, 5 mg/Kg, and 2.5 mg/Kg, respectively. The normal/negative control rats were administered with saline (0.9%).

### 2.4. Animals

Sprague Dawley rats (180–220 g) of either sex were randomly selected and designated as control and treatment groups (seven rats in each group). The animals were kept in aluminum cages (grade 304) under hygienic conditions maintained at 25 ± 2°C with a 12 h light/dark cycle at Primate Facility of Quaid-i-Azam University Islamabad, Pakistan. The rats were well fed and bred in standard conditions with water ad libitum and standard diet. The study design was approved by the Institutional Ethics and Biosafety Committee (BCH = 0275), and all the precautions were carried to minimize animal suffering.

### 2.5. Acute Toxicity Test

To test the acute toxicity of plant extracts a single dose of each extract (400 mg/Kg) was administered orally to all rats in each group. The rats were monitored for any behavioral changes and mortality at interval of 0, 4, 8, 12, 16, and 24 h. The animals were further kept in observation for the next seven days for any symptoms of delayed toxicity or mortality.

### 2.6. Antinociceptive Activity

#### 2.6.1. Hot Plate Assay

Hot plate test was used to determine the antinociceptive activity of selected medicinal plant as reported earlier [38]. Animal were divided into groups having seven rats in each. Group 1 and groups II received normal saline (negative control) and morphine (positive control), respectively. Each remaining group received different plant extract with three different concentrations (200 mg/Kg, 100 mg/Kg, and 50 mg/Kg). Prior to experiment initial latency time (ILT) of animals was measured on hot plate set at 52 ± 1°C and rats having latency time more than 15 s were excluded. After 30 minutes of oral dosage, rats were placed on hot plate under same conditions and data was recorded in the form of licking or jumping response (either comes first). This time was regarded as experimental latency time (ELT). ELT was measured at interval of 0 min, 30 min, 60 min, and 120 min. A cutoff period of 25 s was chosen to avoid tissue damage. Percentage analgesia was measured by following formula.

(1) $$\begin{array}{c} \%\;\;analgesia=\left[\;\left(\;\frac{ELT-ILT}{ILT}\;\right)\times 100\;\right]. \end{array}$$

#### 2.6.2. Writhing Assay

Rat writhing assay was performed by the previously reported method [39] at 200 mg/Kg concentration of extracts. Briefly, animals were divided into controls and experimental groups followed by oral administration of morphine, saline, and plant crude extracts, respectively. After 30 min, each rat was injected intraperitoneally with 0.6% analytical grade acetic acid and rats were housed in a flat glass cylinder. Data was recorded by counting writhing movements which is characterized by abdominal contraction and stretching of hind limbs [40]. Percentage activity was calculated by following formula.

(2) $$\begin{array}{c} \%\;\;analgesia=\left[\;\left(\;\frac{Wc-Ws}{Wc}\;\right)\times 100\;\right], \end{array}$$

where Wc represents mean value of saline group and Ws represents mean value of standard or extract group.

### 2.7. Anti-Inflammatory Activity

#### 2.7.1. Carrageenan Assay

Anti-inflammatory activity of plants was evaluated by carrageenan induced edema test [41]. Sixty minutes before carrageenan injection standard drugs and crude extracts were administered orally. First and second group received saline and diclofenac potassium. Each remaining group was administered with different plant extracts (p.o). Right paw of each rat was injected with 0.1 ml (1%) carrageenan for edema induction. The paw volume was measured before and after the carrageenan induction at 0 h, 1 h, 2 h, 3 h, and 4 h by using Plethysmometer (UGO 7140, Basile) [7]. The percent inhibition of edema was calculated by using the following relation:

(3) $$\begin{array}{c} Percent\;\;edema\;\;inhibition=\left[\;\frac{N.C-S.G}{N.C}\;\right]\times 100, \end{array}$$

where N.C represents negative control group and S.G represents sample group.

#### 2.7.2. Histamine Assay

In histamine assay, edema was induced by injecting 0.1 ml of histamine (1 mg/ml) into the left hind paw of each rat [42]. 1st group received normal saline (negative control; 10 ml/Kg) and 2nd group received chlorpheniramine maleate (positive control; 10 mg/Kg). Each extract was fed orally to separate group (rats = 7) and edema was measured and calculated at 0 min, 30 min, 60 min, 90 min, and 120 min, respectively, as described above in carrageenan assay.

### 2.8. Antidepressant Activity

Antidepressant activity of the extracts was investigated by forced swim assay as reported earlier [38]. Briefly, one day before experiment, preswim test was performed in 21 × 46 cm glass tank filled (30 cm depth) with warm water (35°C). Animals were forced to swim in tank for 10 minutes, dried with towel, and housed back in aluminum cages. On experimental day, animal groups were orally administered with saline, Fluoxetine HCl, and plants extracts, respectively. After 30 minutes animals were released in swim tank and immobility time was calculated.

### 2.9. Anticoagulant Activity

Capillary method was used to investigate the anticoagulant potential of crude extracts as reported by Ismail and Mirza [43]. After 60 minutes of oral administration with saline (negative control), Aspirin (positive control), and crude extracts in respective groups tail of each animal was pierced by sterile lancet. The blood drop was filled into capillary tube. Then a small piece of capillary tube was broken and the same was repeated, till fibrin thread was formed. Time interval between tail pricking and the fibrin formation was recorded.

### 2.10. Statistical Significance Analysis

The data was presented as mean with standard deviation and was analyzed using ANOVA followed by Tukey multiple comparison test. The results were considered to be significant when *p* < 0.05.

## 3. Results and Discussion

### 3.1. Acute Toxicity Test

No mortality was observed for any of the crude extract at 400 mg/K (p.o). Neither of the extracts produces prominent changes in behavior during the time of observation.

### 3.2. Antinociceptive Activity

#### 3.2.1. Hot Plate Assay

**Figure 1:**
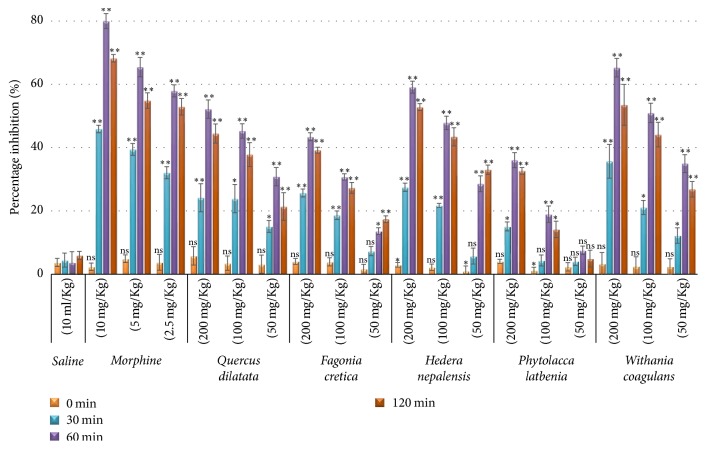
*Percentage inhibition in hot plate assay at different time intervals*. Values expressed in mean ± SD with statistical significance ^*∗*^*p* < 0.05, ^*∗∗*^*p* < 0.01 where “ns” represents nonsignificance.

Hot plate assay is the widely used oldest method to measure nociception in rodents [44]. Latency time at different interval was recorded and presented in Table 1. The extracts showed time and concentration dependent activity which became maximum at 60 min as shown in Figure 1. The results were statistically nonsignificant (*p* < 0.05) at start of the experiment but remained significant at 200 mg and 100 mg/kg with *p* < 0.01. Amongst all,* W. coagulans* extract was more prominent in reducing analgesia with 65.3%  (*p* < 0.01) activity while the extracts of* H. nepalensis* and* Q. dilatata* showed 59.1% and 52.2% activity (Table 3) having *p* value < 0.01.* P. latbenia *and* F. cretica* remained at last with 36% and 43.4% analgesic effect, respectively. This effect was however less than that elicited by morphine (80.0%). Hot plate assay is one of the most suitable and easy methods which represents centrally acting mechanism of pain [45, 46] which generally elevates the pain threshold of rodents to heat or pressure. In this study,* P. latbenia *and* F. cretica *extracts elicited low activity which suggests these extracts do not have centrally acting antinociceptive action.

#### 3.2.2. Writhing Assay

**Figure 2:**
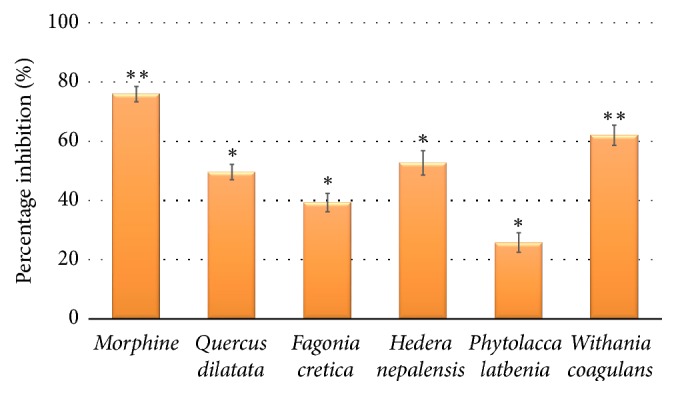
*Percentage inhibition in writhing assay*. Values expressed in mean ± SD with statistical significance ^*∗*^*p* < 0.05, ^*∗∗*^*p* < 0.01.

All crude extracts were subjected to writhing assay at 200 mg/Kg to determine the peripheral antinociceptive activity and results are presented in Figure 2. The most significant activity was detected by* W. coagulans *(62.0%; *p* < 0.01) while* H. nepalensis* displayed 52.7% edema inhibition in statistically significant way (*p* < 0.05). Rest of the plant extracts showed 49.6%, 25.8%, and 39.3% edema inhibition for* Q. dilatata*,* P. latbenia, *and* F. cretica, *respectively (Table 3) with *p* < 0.05. The writhing assay is used for the assessment of peripherally analgesic effect which is characterized by the release of endogenous pain mediators such as arachidonic acid, cyclooxygenase, and prostaglandin [47]. Recently, flavonoids have been reported to halt prostaglandin synthetase [48]. Since prostaglandins are involved in pain perception, it can be suggested that polyphenols present in these plants may be responsible for the observed analgesic activities.

### 3.3. Anti-Inflammatory Activity

#### 3.3.1. Carrageenan Assay

**Figure 3:**
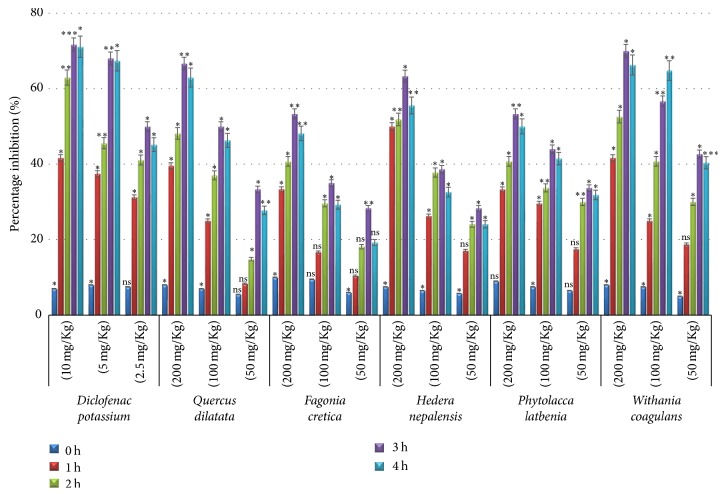
*Percentage inhibition in carrageenan assay at different time intervals*. Values expressed in mean ± SD with statistical significance ^*∗*^*p* < 0.05, ^*∗∗*^*p* < 0.01, and ^*∗∗∗*^*p* < 0.001 where “ns” represents nonsignificance.

Carrageenan assay was used to assess the anti-inflammatory potential of selected medicinal plants. Carrageenan is basically linear and sulfated galactans, isolated from various marine red algae [49]. Results of edema volume measured at different time interval are presented in Table 2 which showed peak edema at 3 h. Percentage edema inhibition was calculated from saline control and presented in Figure 3. The anti-inflammatory effect of diclofenac potassium was found increasing with passage of time being minimum in 1st hour and increased in 2nd hour and remained almost stable in 3rd and 4th hours. The behavior of plant extracts was almost similar as anti-inflammatory activity kept on increasing from first to third hour of injection and a slight decrease in activity was observed at fourth hour in concentration dependent manner (Figure 3). The comparison of the activities (Table 3) showed that the highest percentage activity was exhibited in statistically significant way by* W. coagulans *(70.0%; *p* < 0.05) followed by* Q. dilatata* (66.7%; *p* < 0.01) and* H. nepalensis* (63.3%; *p* < 0.05) while* P. latbenia *and* F. cretica *displayed almost same level of edema inhibition (53.3%; *p* < 0.01). Carrageenan assays are generally reported as a suitable model to study anti-inflammatory properties of medicinal plants as they involve several mediator molecules [50]. The inflammation process has been proposed in three phases including 1st phase (0–1.5 h) required for the action of mediators such as serotonin and histamine, 2nd phase (1.5–2.5 h) facilitated by bradykinin, and 3rd phase (2.5–6 h) mediated by release of prostaglandins [51]. Our results showed significant inhibition in the 1st phase which suggests that the extracts may also inhibit the release of histamine.

#### 3.3.2. Histamine Assay

**Figure 4:**
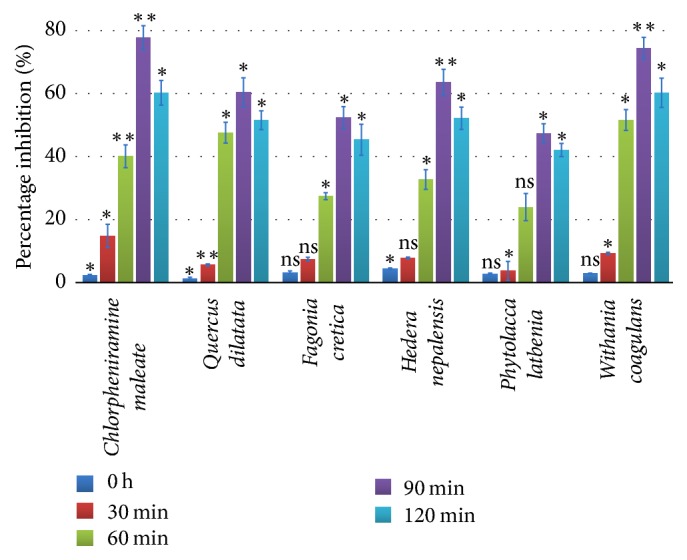
*Percentage inhibition in histamine assay at different time intervals*. Values expressed in mean ± SD with statistical significance ^*∗*^*p* < 0.05, ^*∗∗*^*p* < 0.01 where “ns” represents nonsignificance.

In order to confirm the findings of carrageenan assay, the crude extracts at the most effective concentration (200 mg/Kg) of each plant were evaluated by using histamine edema model. The maximum edema inhibition was detected at 90 min (Figure 4). The most significant activity was observed by* W. coagulans *(74.3%; *p* < 0.01) while chlorpheniramine maleate (positive control) displayed 77% (*p* < 0.01) edema inhibition.* Q. dilatata* (60.4%; *p* < 0.05) and* H. nepalensis* (63.5%; *p* < 0.01) showed moderate activity while* P. latbenia *and* F. cretica *exhibited 47.4% and 52.4% activity accordingly (Table 3) with statistical significance (*p* < 0.05). The results exhibited that the plant extracts have effectively reduced the inflammation produced by histamine which indicates that the extracts exhibit anti-inflammatory action by inhibiting the release of histamine.

### 3.4. Antidepressant Activity

**Figure 5:**
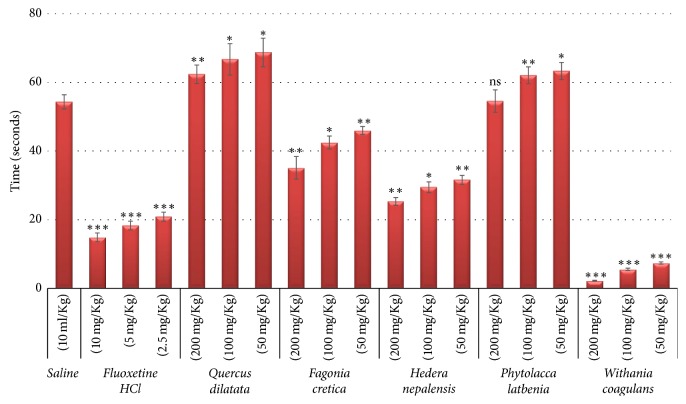
*Results of antidepressant assay*. Values expressed in mean ± SD with statistical significance ^*∗*^*p* < 0.05, ^*∗∗*^*p* < 0.01, and ^*∗∗∗*^*p* < 0.001 where “ns” represents nonsignificance.

The depression in force swim test is categorized by increase in immobility and decrease in swimming [52]. When rats were forced to swim, they quickly stopped swimming and held still. This behavior was designated as immobility and showed depressed mood. The mediators that reduce this behavior are known as antidepressant [53]. The force swim test is highly specific as depression is decreased by numerous agents like tricyclics, serotonin reuptake, and MAO inhibitors [54]. Antidepressant activities of selected medicinal plants were tested by forced swim assay at three different concentration (200, 100, and 50 mg/Kg) and results in the form of immobility time are presented in Figure 5. Fluoxetine HCl and saline control showed prominent difference in mobility time (54.3 s and 14.9 s, resp.) which represent the reliability of assay. The most prominent activity was exhibited by* W. coagulans *with immobility times 2.2 s, 5.5 s, and 7.4 s with decreasing concentration in a highly significant manner (*p* < 0.001). Approximately 95.9% activity was exhibited by the* W. coagulans *while positive control (Fluoxetine HCl) showed 72%  (*p* < 0.001) activity as compared with saline control (Table 3).* F. cretica* and* H. nepalensis* showed moderate antidepressant activity with immobility times 35.1 s and 25.3 s, respectively, at 200 mg/Kg with *p* < 0.01. Nonsignificant activity (*p* > 0.05) was shown by* Q. dilatata* and* P. latbenia* as their immobility times were 62.4 s and 54.6 s at the highest concentration accordingly. Overall results showed a significant decrease in immobility time in a concentration dependent manner.* W. coagulans, F. cretica,* and* H. nepalensis *are known to have different flavonoid and phenolic compounds with several biological activities in central nerves system. Although the role of flavonoids and phenolics in these plants has not been defined as antidepressant, however these compounds are well known for antidepressant effect [7, 55, 56]. So, it can be suggested that these compounds may be responsible for antidepressant effect.

### 3.5. Anticoagulant Activity

**Figure 6:**
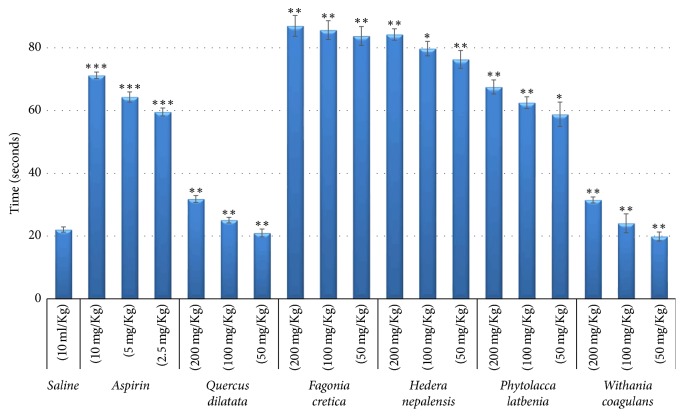
*Results of anticoagulant assay*. Values expressed in mean ± SD with statistical significance ^*∗*^*p* < 0.05, ^*∗∗*^*p* < 0.01, and ^*∗∗∗*^*p* < 0.001.

Anticoagulant activity of selected medicinal plants was determined by capillary method. Experiments were performed at 200, 100, and 50 mg/Kg concentrations and results are given in Figure 6. Aspirin (positive control) and saline (negative control) had blood coagulation times 71.2 s and 22.1 s, respectively, which draws a clear line of difference between the anticoagulation and coagulation. Prominent and significant (*p* < 0.01) anticoagulant activity was presented by* F. cretica* and* H. nepalensis* with coagulation times of 86.9 s and 84.2 s, respectively, at 200 mg/Kg. However, at 100 mg/Kg coagulation times were 85.6 s and 79.7 s while at 50 mg/Kg the times were 83.8 s and 76.3 s accordingly.* P. latbenia* showed moderate but significant (*p* < 0.05) antidepressant activity with coagulation times 67.5 s, 62.8 s, and 58.8 s, respectively, with decreasing concentrations. Roughly,* F. cretica*,* H. nepalensis,* and* P. latbenia* showed 74.6%, 73.8%, and 67.3% enhanced activity with *p* value less than 0.01 as compared with saline control (0%) while Aspirin exhibited 69.0%  (*p* < 0.001) activity (Table 3). The blood coagulation time of* Q. dilatata* (31.8 s; *p* < 0.01) and* W. coagulans* (31.5 s; *p* < 0.01) was about equal to saline control (22.1 s) representing neutral behavior of these plant extracts. Coagulation of blood can be delayed by numerous agents in blood, having different mode of actions. Most of them depend on the inhibition of clotting factors which are major cause of heart attacks and strokes [57]. Activation of antithrombin-III is the one the mechanisms and anticoagulant drugs like Lepirudin, Dalteparin, and Fondaparinux are the other [58, 59]. To cope with such problems herbal products or medicines can be used instead of blood thinners. The promising anticoagulant effect of* F. cretica*,* H. nepalensis,* and* P. latbenia* opens the doors for the further mechanism of action study in herbal medicines.

## 4. Conclusion

The hot plate assay is a simple method of the pain reaction in animals by which effectiveness of analgesics can be tested by measuring the heat induced pain response. Conversely, if a condition such as an inflammation of the paw serves to decrease the response latency, it is said to induce hyperalgesia which means these assays have some positive correlation between them. In contrast forced swim test and anticoagulant assays are unrelated so no correlation can be found between them. Forced swimming test represents the pharmacological model and produces a state similar to human depression which is very specific and sensitive while anticoagulant assay deals with the hemostatic mechanisms.

From the results of biological screening on rats it was concluded that* Q. dilatata* showed moderate activity in ant-inflammatory and analgesic assay. The crude extract of* F. cretica* exhibited prominent activity in anticoagulant assay while* P. latbenia* extracts showed moderate activity only in antidepressant assay.* H. nepalensis* showed positive results in all assays which represents the multidrug potential of this plant. As far as the extract of* W. coagulans *is concerned most promising results were exhibited by this plant in terms of in anti-inflammatory, analgesic, and antidepressant property. So, overall comparison of all the plant extracts showed that* W. coagulans* and* H. nepalensis* are the most gifted plants and have good potential for further studies.

## Figures and Tables

**Table 1:** Latency time in hot plate assay.

Treatment	Dose	^¥^Latency time (sec)			
		0 min	30 min	60 min	120 min
Saline	10 ml/Kg	4.7 ± 0.06	4.7 ± 0.1	4.6 ± 0.15	4.8 ± 0.06

Morphine	10 mg/Kg	8.7 ± 0.10^*∗∗*^	12.4 ± 0.10^*∗∗*^	15.3 ± 0.20^*∗*^	14.3 ± 0.10^*∗∗*^
	5 mg/Kg	8.6 ± 0.10^*∗∗*^	11.4 ± 0.15^*∗∗*^	13.6 ± 0.25^*∗*^	12.7 ± 0.20^*∗*^
	2.5 mg/Kg	8.3 ± 0.20^*∗*^	10.6 ± 0.15^*∗∗*^	12.6 ± 0.15^*∗∗*^	12.2 ± 0.21^*∗*^

*Quercus dilatata*	200 mg/Kg	7.3 ± 0.20^*∗*^	8.6 ± 0.31^*∗*^	10.5 ± 0.20^*∗*^	10.0 ± 0.21^*∗*^
	100 mg/Kg	6.9 ± 0.15^*∗∗*^	8.3 ± 0.30^*∗*^	9.7 ± 0.15^*∗∗*^	9.2 ± 0.25^*∗*^
	50 mg/Kg	5.5 ± 0.15^*∗∗*^	6.1 ± 0.10^*∗∗*^	6.9 ± 0.15^*∗*^	6.4 ± 0.23^*∗*^

*Fagonia cretica*	200 mg/Kg	17.2 ± 0.15^*∗∗*^	20.7 ± 0.21^*∗*^	23.7 ± 0.20^*∗*^	23.0 ± 0.15^*∗∗*^
	100 mg/Kg	16.0 ± 0.20^*∗*^	18.3 ± 0.21^*∗*^	20.1 ± 0.15^*∗∗*^	19.6 ± 0.26^*∗*^
	50 mg/Kg	14.4 ± 0.21^*∗*^	15.2 ± 0.21	16.1 ± 0.15^*∗∗*^	16.7 ± 0.15^*∗∗*^

*Hedera nepalensis*	200 mg/Kg	16.4 ± 0.12^*∗∗*^	20.3 ± 0.21^*∗*^	25.3 ± 0.30^*∗*^	24.3 ± 0.17^*∗∗*^
	100 mg/Kg	15.5 ± 0.15^*∗∗*^	18.5 ± 0.10^*∗∗*^	22.5 ± 0.32^*∗*^	21.8 ± 0.44^*∗*^
	50 mg/Kg	10.6 ± 0.17^*∗*^	11.1 ± 0.26^*∗*^	13.5 ± 0.26^*∗*^	14.0 ± 0.15^*∗∗*^

*Phytolacca latbenia*	200 mg/Kg	15.4 ± 0.10^*∗∗*^	17.0 ± 0.21^*∗*^	20.1 ± 0.35^*∗*^	19.6 ± 0.15^*∗∗*^
	100 mg/Kg	14.8 ± 0.15^*∗∗*^	15.2 ± 0.25^*∗*^	17.4 ± 0.38^*∗*^	16.7 ± 0.38^*∗*^
	50 mg/Kg	11.8 ± 0.15^*∗∗*^	12.0 ± 0.15^*∗∗*^	12.4 ± 0.15^*∗∗*^	12.1 ± 0.31^*∗*^

*Withania coagulans*	200 mg/Kg	7.3 ± 0.25^*∗*^	9.6 ± 0.38^*∗*^	11.7 ± 0.21^*∗*^	10.9 ± 0.46^*∗*^
	100 mg/Kg	7.0 ± 0.21^*∗*^	8.3 ± 0.15^*∗∗*^	10.3 ± 0.21^*∗*^	9.8 ± 0.26^*∗*^
	50 mg/Kg	4.2 ± 0.10^*∗∗*^	4.6 ± 0.10^*∗∗*^	5.5 ± 0.12^*∗∗*^	5.2 ± 0.10^*∗∗*^

^*¥*^Mean values with SD expressed in ^*∗*^*p* < 0.05, ^*∗∗*^*p* < 0.01 statistical significance compared with saline group.

**Table 2:** Edema volume of rat's paw in carrageenan assay.

Treatment	Dose	^¥^Absolute edema volume (ml)			
		1 h	2 h	3 h	4 h
Saline	10 ml/Kg	0.24 ± 0.02	0.27 ± 0.03	0.30 ± 0.01	0.27 ± 0.02

Diclofenac potassium	10 mg/Kg	0.14 ± 0.005^*∗∗*^	0.10 ± 0.003^*∗∗∗*^	0.08 ± 0.02^*∗*^	0.07 ± 0.01^*∗*^
	5 mg/Kg	0.15 ± 0.06^*∗*^	0.14 ± 0.002^*∗∗*^	0.09 ± 0.05^*∗*^	0.08 ± 0.02^*∗*^
	2.5 mg/Kg	0.16 ± 0.04^*∗*^	0.16 ± 0.05^*∗*^	0.15 ± 0.01^*∗*^	0.14 ± 0.03^*∗*^

*Quercus dilatata*	200 mg/Kg	0.14 ± 0.03^*∗*^	0.14 ± 0.04^*∗*^	0.10 ± 0.02^*∗*^	0.10 ± 0.02^*∗*^
	100 mg/Kg	0.18 ± 0.02^*∗*^	0.17 ± 0.03^*∗*^	0.15 ± 0.005^*∗∗*^	0.14 ± 0.05^*∗*^
	50 mg/Kg	0.22 ± 0.05^*∗*^	0.23 ± 0.02^*∗*^	0.20 ± 0.06^*∗*^	0.19 ± 0.01^*∗*^

*Fagonia cretica*	200 mg/Kg	0.16 ± 0.05^*∗*^	0.16 ± 0.06^*∗*^	0.14 ± 0.02^*∗*^	0.14 ± 0.005^*∗*^
	100 mg/Kg	0.20 ± 0.06^*∗*^	0.19 ± 0.02^*∗*^	0.20 ± 0.04^*∗*^	0.19 ± 0.05^*∗*^
	50 mg/Kg	0.21 ± 0.04^*∗*^	0.22 ± 0.02^*∗*^	0.21 ± 0.05^*∗*^	0.22 ± 0.03^*∗*^

*Hedera nepalensis*	200 mg/Kg	0.12 ± 0.01^*∗*^	0.13 ± 0.01^*∗*^	0.11 ± 0.05^*∗*^	0.12 ± 0.02^*∗*^
	100 mg/Kg	0.17 ± 0.02^*∗*^	0.16 ± 0.03^*∗*^	0.18 ± 0.06^*∗*^	0.18 ± 0.01^*∗*^
	50 mg/Kg	0.19 ± 0.02^*∗*^	0.20 ± 0.04^*∗*^	0.21 ± 0.03^*∗*^	0.20 ± 0.03^*∗*^

*Phytolacca latbenia*	200 mg/Kg	0.16 ± 0.02^*∗*^	0.16 ± 0.05^*∗*^	0.14 ± 0.02^*∗*^	0.13 ± 0.05^*∗*^
	100 mg/Kg	0.16 ± 0.02^*∗*^	0.18 ± 0.04^*∗*^	0.17 ± 0.05^*∗*^	0.16 ± 0.04^*∗*^
	50 mg/Kg	0.20 ± 0.03^*∗*^	0.19 ± 0.01^*∗*^	0.20 ± 0.04^*∗*^	0.18 ± 0.03^*∗*^

*Withania coagulans*	200 mg/Kg	0.14 ± 0.01^*∗*^	0.13 ± 0.01^*∗*^	0.09 ± 0.04^*∗*^	0.09 ± 0.05^*∗*^
	100 mg/Kg	0.18 ± 0.06^*∗*^	0.16 ± 0.05^*∗*^	0.13 ± 0.03^*∗*^	0.09 ± 0.04^*∗*^
	50 mg/Kg	0.20 ± 0.05^*∗*^	0.19 ± 0.06^*∗*^	0.17 ± 0.04^*∗*^	0.16 ± 0.02^*∗*^

^*¥*^Calculated from respective hour paw volume – 0 hour paw volume. Expressed as mean ± SD with ^*∗*^*p* < 0.05, ^*∗∗*^*p* < 0.01, and ^*∗∗∗*^*p* < 0.001 statistical significance compared with saline group.

**Table 3:** Comparison of five plant activities in different assays.

Crude plant extract	Percentage Activity					
	Analgesic assays^¥^		Antidepressant assay^§^	Anticoagulant assay^§^	Anti-inflammatory assays^¥^	
	Hot plate	Writhing			Carrageenan	Histamine
*Quercus dilatata*	52.2%	49.6%	0%	30.1%	66.7%	60.4%
*Fagonia cretica*	43.4%	39.3%	35.4%	74.6%	53.3%	52.4%
*Hedera nepalensis*	59.1%	52.7%	53.4%	73.8%	63.3%	63.5%
*Phytolacca latbenia*	36.0%	25.8%	0%	67.3%	53.3%	47.4%
*Withania coagulans*	65.3%	62.0%	95.9%	29.8%	70.0%	74.3%

^**¥**^Expressed as mean of highest activity. ^**§**^Calculated from negative control group.

## References

[1] Ullah N. (2017). Medicinal plants of Pakistan: Challenges and Opportunities. *Journal of Complementary & Alternative Medici*.

[2] Cragg G. M., Newman D. J., Snader K. M. (1997). Natural products in drug discovery and development. *Journal of Natural Products*.

[3] Pan S.-Y., Zhou S.-F., Gao S.-H. (2013). New perspectives on how to discover drugs from herbal medicines: CAM'S outstanding contribution to modern therapeutics. *Evidence-Based Complementary and Alternative Medicine*.

[4] Azaizeh H., Saad B., Khalil K., Said O. (2006). The state of the art of traditional Arab herbal medicine in the Eastern region of the Mediterranean: a review. *Evidence-Based Complementary and Alternative Medicine*.

[5] Yin S.-Y., Wei W.-C., Jian F.-Y., Yang N.-S. (2013). Therapeutic applications of herbal medicines for cancer patients. *Evidence-Based Complementary and Alternative Medicine*.

[6] Sajid M., Khan M. R., Shah N. A. (2016). Phytochemical, antioxidant and hepatoprotective effects of Alnus nitida bark in carbon tetrachloride challenged Sprague Dawley rats. *BMC Complementary and Alternative Medicine*.

[7] Dilshad E., Ismail H., Haq I.-U. (2016). Rol genes enhance the biosynthesis of antioxidants in Artemisia carvifolia Buch. *BMC Plant Biology*.

[8] Ahmed E., Arshad M., Ahmad M., Saeed M., Ishaque M. (2004). Ethnopharmacological survey of some medicinally important plants of Galliyat Areas of NWFP, Pakistan. *Asian Journal of Plant Sciences*.

[9] Shah S. (2005). Pollen morphology of three species of quercus (Family Fagaceae) [Quercus dilatata, Q. incana and Q. ballota]. *Journal of Agriculture and Social Sciences*.

[10] Haq F., Ahmad H., Alam M. (2011). Traditional uses of medicinal plants of Nandiar Khuwarr catchment (District Battagram), Pakistan. *Journal of Medicinal Plants Research*.

[11] Ihtisham M., Ihsan-Ul-Haq, Sarwar S., Mirza B. (2013). HPLC-DAD analysis and free radical scavenging potential of Quercus dilatata L. *Pakistan Journal of Botany*.

[12] Umachigi S. P., Jayaveera K. N., Ashok Kumar C. K., Kumar G. S., Vrushabendra swamy B. M., Kishore Kumar D. V. (2008). Studies on Wound Healing Properties of Quercus infectoria. *Tropical Journal of Pharmaceutical Research*.

[13] Hussain A., Zia M., Mirza B. (2007). Cytotoxic and antitumor potential of Fagonia cretica L. *Turkish Journal of Biology*.

[14] Baquar S. R. (1989). *Medicinal and poisonous plants of Pakistan*.

[15] Said H. M. (1969). Hammardad pharmacopoeia of Easteren Medicine. *Institute of Health and Tibbi Research*.

[16] Tahir Razi M., Asad M. H. H. B., Khan T. (2011). Antihaemorrhagic potentials of Fagonia cretica against Naja naja karachiensis (black Pakistan cobra) venom. *Natural Product Research (Formerly Natural Product Letters)*.

[17] Singh U., Wadhwani A., Johri B. (1983). Dictionary of economic plants in India. *Dictionary of Economic Plants in India*.

[18] Kasture V. S., Gosavi S. A., Kolpe J. B., Deshapande S. G. (2014). Phytochemical and biological evaluation of Fagonia species: a review. *World Journal of Pharmacy and Pharmaceutical Sciences*.

[19] Said H. M., Saeed A. (1996). *Medicinal herbal a textbook for medical students and doctors*.

[20] Ahmad S. S. (2007). Medicinal wild plants from Lahore-Islamabad motorway (M-2). *Pakistan Journal of Botany*.

[21] Bashir A., Nida M., Shumaila B., Sadiq A., Ibrar K., Mohammad A. (2012). Biological screening of Hedera nepalensis. *Journal of Medicinal Plants Research*.

[22] Hamayun M., Khan S. A., Sohn E. Y., Lee I.-J. (2006). Folk medicinal knowledge and co nservation status of some economically valued medicinal plants of district Swat, Pakistan. *Lyonia*.

[23] Qureshi R. A., Ghufran M. A., Gilani S. A., Yousaf Z., Abbas G., Batool A. (2009). Indigenous medicinal plants used by local women in southern Himalayan regions of Pakistan. *Pakistan Journal of Botany*.

[24] Qureshi R. A., Ghufran M. A., Gilani S. A., Sultana K., Ashraf M. (2007). Ethnobotanical studies of selected medicinal plants of sudhan gali and ganga chotti hills, district bagh, azad kashmir. *Pakistan Journal of Botany*.

[25] Gilani S. A., Qureshi R. A., Gilani S. J. (2006). Indigenous uses of some important ethnomedicinal herbs of Ayubia National Park, Abbottabad, Pakistan. *Ethnobotanical Leaflets*.

[26] Shah G. M., Khan M. A. (2006). Checklist of Medicinal Plants of Siran Valley, Mansehra, Pakistan. *Ethnobotanical Leaflets*.

[27] Ullah N., Haq I. U., Safdar N., Mirza B. (2015). Physiological and biochemical mechanisms of allelopathy mediated by the allelochemical extracts of Phytolacca latbenia (Moq.) H. Walter. *Toxicology & Industrial Health*.

[28] Ullah N., Haq I.-U., Mirza B. (2015). Phytotoxicity evaluation and phytochemical analysis of three medicinally important plants from Pakistan. *Toxicology & Industrial Health*.

[29] Yong O. K., Johnson J. D., Eun J. L. (2005). Phytotoxic effects and chemical analysis of leaf extracts from three Phytolaccaceae species in South Korea. *Journal of Chemical Ecology*.

[30] Tareen R. B., Bibi T., Khan M. A., Ahmad M., Zafar M. (2010). Indigenous knowledge of folk medicine by the women of Kalat and Khuzdar regions of Balochistan, Pakistan. *Pakistan Journal of Botany*.

[31] Gupta P. C. (2012). Withania coagulans Dunal- An overview. *International Journal of Pharmaceutical Sciences Review and Research*.

[32] Naz S., Masud T., Nawaz M. A. (2009). Characterization of milk coagulating properties from the extract of Withania coagulans. *International Journal of Dairy Technology*.

[33] Ullah M., Khan M. U., Mahmood A. (2013). An ethnobotanical survey of indigenous medicinal plants in Wana district south Waziristan agency, Pakistan. *Journal of Ethnopharmacology*.

[34] Gupta V., Keshari B. B. (2013). Withania coagulans Dunal (paneer doda): A review. *International Journal of Ayurvedic and Herbal Medicine*.

[35] Maurya R., Akanksha, Jayendra (2010). Chemistry and pharmacology of Withania coagulans: An Ayurvedic remedy. *Journal of Pharmacy and Pharmacology*.

[36] Atta-ur-Rahman, Dur-e-Shahwar, Naz A., Choudhary M. I. (2003). Withanolides from Withania coagulans. *Phytochemistry*.

[37] Khodaei M., Jafari M., Noori M. (2012). Remedial Use of Withanolides from Withania Coagolans (Stocks) Dunal. *Advances in Life Sciences*.

[38] Ismail H., Dilshad E., Waheed M. T., Sajid M., Kayani W. K., Mirza B. (2016). Transformation of Lactuca sativa L. with rol C gene results in increased antioxidant potential and enhanced analgesic, anti-inflammatory and antidepressant activities in vivo. *3 Biotech*.

[39] Abdollahi M., Karimpour H., Monsef-Esfehani H. R. (2003). Antinociceptive effects of Teucrium polium L. total extract and essential oil in mouse writhing test. *Pharmacological Research*.

[40] Jain S. C., Jain R., Sharma R. A., Capasso F. (1997). Pharmacological investigation of Cassia italica. *Journal of Ethnopharmacology*.

[41] Ismail H., Dilshad E., Waheed M. T., Mirza B. (2017). Transformation of Lettuce with rol ABC Genes: Extracts Show Enhanced Antioxidant, Analgesic, Anti-Inflammatory, Antidepressant, and Anticoagulant Activities in Rats. *Applied Biochemistry and Biotechnology*.

[42] Sajid M., Khan M. R., Shah S. A. (2017). Investigations on anti-inflammatory and analgesic activities of Alnus nitida Spach (Endl). stem bark in Sprague Dawley rats. *Journal of Ethnopharmacology*.

[43] Ismail H., Mirza B. (2015). Evaluation of analgesic, anti-inflammatory, anti-depressant and anti-coagulant properties of Lactuca sativa (CV. Grand Rapids) plant tissues and cell suspension in rats. *BMC Complementary and Alternative Medicine*.

[44] Gunn A., Bobeck E. N., Weber C., Morgan M. M. (2011). The influence of non-nociceptive factors on hot-plate latency in rats. *The Journal of Pain*.

[45] Khan H., Saeed M., Gilani A. U. H., Khan M. A., Khan I., Ashraf N. (2011). Antinociceptive activity of aerial parts of *Polygonatum verticillatum*: attenuation of both peripheral and central pain mediators. *Phytotherapy Research*.

[46] Yaseen T., Ibrar M., Barkatullah., Muhammad N. (2013). Antinociceptive and laxative profile of Withania coagulans leaves. *Medicinal Chemistry & Drug Discovery*.

[47] Franzotti E. M., Santos C. V. F., Rodrigues H. M. S. L., Mourão R. H. V., Andrade M. R., Antoniolli A. R. (2000). Anti-inflammatory, analgesic activity and acute toxicity of *Sida cordifolia* L. (Malva-branca). *Journal of Ethnopharmacology*.

[48] Hugar M. H., Hosamani K. M., Liyakath Ahmed M. D. (2010). Phytochemical and pharmacological studies of ethanol extract of Dalbergia sissoo seeds: An approach for the in-vivo analgesic and antipyretic activities. *International Journal of Pharma and Bio Sciences*.

[49] Navarro D. A., Stortz C. A. (2005). Microwave-assisted alkaline modification of red seaweed galactans. *Carbohydrate Polymers*.

[50] Mequanint W., Makonnen E., Urga K. (2011). In vivo anti-inflammatory activities of leaf extracts of Ocimum lamiifolium in mice model. *Journal of Ethnopharmacology*.

[51] di Rosa M., Giroud J. P., Willoughby D. A. (1971). Studies on the mediators of the acute inflammatory response induced in rats in different sites by carrageenan and turpentine. *The Journal of Pathology*.

[52] Kirby L. G., Lucki I. (1997). Interaction between the forced swimming test and fluoxetine treatment on extracellular 5-hydroxytryptamine and 5-hydroxyindoleacetic acid in the rat. *The Journal of Pharmacology and Experimental Therapeutics*.

[53] Porsolt R. D., Bertin A., Blavet N., Deniel M., Jalfre M. (1979). Immobility induced by forced swimming in rats: Effects of agents which modify central catecholamine and serotonin activity. *European Journal of Pharmacology*.

[54] Detke M. J., Rickels M., Lucki I. (1995). Active behaviors in the rat forced swimming test differentially produced by serotonergic and noradrenergic antidepressants. *Psychopharmacology*.

[55] Velioglu Y., Mazza G. (1991). Characterization of flavonoids in petals of Rosa damascena by HPLC and spectral analysis. *Journal of Agricultural and Food Chemistry*.

[56] Moallem S. A., Hosseinzadeh H., Ghoncheh F. (2007). Evaluation of antidepressant effects of aerial parts of Echium vulgare on mice. *Iranian Journal of Basic Medical Sciences*.

[57] Meade T. W., Ruddock V., Stirling Y., Chakrabarti R., Miller G. J. (1993). Fibrinolytic activity, clotting factors, and long-term incidence of ischaemic heart disease in the Northwick Park Heart Study. *The Lancet*.

[58] Chackalamannil S. (2006). Thrombin receptor (protease activated receptor-1) antagonists as potent antithrombotic agents with strong antiplatelet effects. *Journal of Medicinal Chemistry*.

[59] Moll S., Roberts H. R. (2002). Overview of anticoagulant drugs for the future. *Seminars in Hematology*.

